# Validity and reliability of the Amsterdam Preoperative Anxiety and Information Scale in the Turkish population

**DOI:** 10.3906/sag-1806-84

**Published:** 2019-02-11

**Authors:** Funda ÇETİNKAYA, Esin KAVURAN, Kevser Sevgi ÜNAL ASLAN

**Affiliations:** 1 Department of Surgical Nursing, Faculty of Health Sciences, Aksaray University, Aksaray Turkey; 2 Faculty of Nursing, Atatürk University, Erzurum Turkey; 3 Department of Nursing, School of Health, Osmaniye Korkut Ata University, Osmaniye Turkey

**Keywords:** Patients, preoperative anxiety, reliability, validity

## Abstract

**Background/aim:**

This study aimed to adapt the Amsterdam Preoperative Anxiety and Information Scale (APAIS) to measure the preoperative anxiety of adult Turkish patients undergoing surgery.

**Materials and methods:**

The sample of this methodological study included 210 patients. Data were collected by using personal information forms, the APAIS, and the State Anxiety Scale (STAI). Cronbach’s α-coefficient was calculated, and test–retest reliability was tested.

**Results:**

Cronbach’s α-coefficients of the APAIS anxiety and information requirement subscales were 0.897 and 0.786, respectively. The mean test and retest scores of the APAIS were not different. The test and retest scores of the patients were significantly, positively, and strongly related. The APAIS and STAI-I were correlated. Factorial analysis revealed that two factors accounted for 81.435% of the total variance with an eigenvalue of >1. These results showed that the Turkish version of the APAIS is a valid and reliable scale.

**Conclusion:**

The Turkish translation of the APAIS is valid and can be reliably used to determine the preoperative anxiety experienced by patients who are undergoing elective surgery.

## 1. Introduction

Anxiety is a disturbing feeling of fear and concern that is perceived as life-threatening (1). Anxiety, the feeling of restlessness and tension caused by the expectation of danger, can cause numerous physiological and psychological problems by increasing sympathetic, parasympathetic, and endocrine stimuli (2). Preoperative anxiety is a globally encountered problem in the healthcare field and is defined as fear that is experienced by patients who will undergo surgery (3). Most patients experience different degrees of anxiety and fear before surgery (4). The causes of preoperative anxiety include waking up during surgery; failure to wake up after surgery: postoperative pain; nausea and vomiting; potential stay in intensive care; incompetent, inexperienced, or absent anesthetist; fear of needles, death, or incomprehensible babbling under anesthesia; and pain during surgery (3,5,6). High levels of preoperative anxiety cause physical problems, such as dizziness, nausea, and headache, and affect postoperative anxiety. Moreover, high levels of preoperative anxiety increase the anesthesia dosage required during surgery and the analgesic dosage required for postoperative pain management (4) and adversely affect cognitive functions (7). Effective preoperative patient assessment and relieving the anxieties of extremely anxious patients through appropriate nursing interventions are necessary for patients to experience problem-free postoperative periods and short hospital stays.

Various methods are used to decrease the anxiety levels of patients. These methods include preoperative interviews with the anesthesiologist and nurses, as well as information briefing (1,8,9). Various tools are used to assess the preoperative anxiety levels of patients. One of the most commonly used instruments for measuring anxiety is Spielberger’s State Trait Anxiety Inventory (STAI). The STAI is frequently used to determine the anxiety levels of patients in Turkey given its proven validity and reliability in Turkish society (10). However, the need for clinically practical and rapid assessment tools for anxiety levels may sometimes arise. Moerman et al. developed the Amsterdam Preoperative Anxiety and Information Scale (APAIS) to assess the preoperative anxiety and information requirements of patients (11). The APAIS was originally developed in the Netherlands but has since been translated into valid and reliable English (12), Japanese (13), Italian (14), German (15), Spanish (16), and French (17) versions.

This study was conducted to evaluate the validity and reliability of the Turkish translation of the APAIS. 

## 2. Materials and methods

### 2.1. Design

The study was conducted as a methodological design in order to evaluate the psychometric characteristics of the Turkish version of the APAIS. The first step was the translation of the APAIS into Turkish. The translation was made by bilingual authors according to existing guidelines and back-translations were made to guarantee the maximum adherence to the original version (18–20). The pilot test involved 10 patients (five women and five men). The questionnaire was well received and patients did not report any problems in answering the questions. 

### 2.2. Study participants

This methodological study was conducted to adapt and investigate the validity and reliability of the APAIS for Turkish adult patients who were undergoing surgery. The study data were collected over the period of February–April 2018. In the literature, it is determined that the number of samples is to be 5–10 times greater than the number of items in scale adaptation studies (21). During a period of 3 months, 400 patients visited the surgery clinics of a training and research hospital in Aksaray and 190 patients who did not meet the study criteria were excluded, so the study sample consisted of 210 patients who were selected in accordance with the following criteria: >18 years old; fully coherent and conscious; lacking vision, hearing, or motor-skill problems; and able to read, write, speak, and understand Turkish.

### 2.3. Ethical considerations

Permission to translate the APAIS into Turkish and to use it in this research was obtained via e-mail from Moerman, who developed the APAIS. The Ethical Committee of Aksaray University approved this study (Protocol No: 2017/104). The recruited patients provided verbal and written consent. During data gathering, questions from the participants were answered. 

### 2.4. Data collection 

Research data were collected 24–48 h prior to surgery through onsite face-to-face interviews. Patients completed the APAIS scale in approximately 3 min. The average duration required to complete all questionnaire forms was 25 min.

Study data were collected by using personal information forms, the APAIS, and the STAI. 

#### 2.4.1. Personal information form

The form contains 10 items and was developed by the researchers. The form includes questions about the patient’s basic information, such as age, sex, marital status, occupation, and chronic disease type.

#### 2.4.2. APAIS

The APAIS is a six-item questionnaire used to for the rapid assessment of preoperative anxiety. The APAIS consists of two scales that include a four-item anxiety scale and a two-item information requirement scale. The items are rated on a Likert scale from 1 (“not at all worrying”) to 5 (“extremely worrying”) (5). The score ranges of the anxiety subscale and information requirement subscale are 4–20 and 2–10, respectively. High scores are associated with high anxiety levels and information requirement. The Cronbach’s α-coefficients for the anxiety subscale and information requirement subscale were 0.86 and 0.68, respectively (11).

#### 2.4.3. STAI

The STAI was developed by Spielberg et al. in the USA in 1970. It was adapted and validated for use in Turkey by Öner and Le Compte. The STAI comprises 40 items subdivided into STAI-I, a 20-item self-reported rating scale for trait measurement, and STAI-II, a 20-item for reporting anxiety state (10). 

### 2.5. Data analysis

Data were analyzed using SPSS for Windows. The internal consistency of the scales was evaluated on the basis of Cronbach’s α-coefficient. Construct validity was evaluated through factorial analysis. Spearman’s coefficients were calculated to explore the correlation between the APAIS and STAI. KMO coefficients and Bartlett’s test results were observed to determine whether the dataset was fit for factorial analysis.

## 3. Results

The mean age of the 210 patients (98 [46.7%] females, 112 [53.3%] males) included in the study was 50.16 ± 17.96 years. The demographics and operation types of the patients are presented in Table 1.

**Table 1:** Sociodemographic characteristics of participants.

Characteristics		
Age (mean ± SD)	50.16 ± 17.96	
	N	(%)
Sex		
Female	98	46.7
Male	112	53.3
Marital status		
Married	165	78.6
Single	45	21.4
Education status		
Not literate	42	20.0
Primary education	122	58.1
High school	31	14.8
University	15	7.3
Occupation		
Officer	18	8.6
Laborer	30	14.3
Self-employed	42	20.0
Retired	28	13.3
Housewife	81	38.6
Student	11	5.2
Place of residence		
Provincial center	123	58.6
District	40	19.0
Village	47	22.4
Health insurance		
Yes	193	91.9
No	17	8.1
Chronic disease		
Yes	89	42.4
No	121	57.6
Uses drugs		
Yes	100	47.6
No	110	52.4
Previous surgery		
Yes	133	63.3
No	77	36.7
Clinics		
General surgery	59	28.1
Brain surgery	32	15.2
Cardiovascular surgery	18	8.6
Urology	19	9.0
Orthopedics	35	16.7
Plastic surgery	34	16.2
ENT	12	5.7
Eye	1	0.5
Operation type		
Major	27	12.9
Middle	67	31.9
Minor	116	55.2
Anesthetic type		
General anesthesia	99	47.1
Local anesthesia	111	52.9
Total	210	100.0

Two independent forward translations from Dutch to Turkish and English were obtained and merged into a single tool by three academics with a good command of the Dutch language. The tool was back-translated into Dutch, which is the original language of the scale, by a bilingual translator who has a good command of Turkish and Dutch. The back-translated scale was compared with the original Dutch and translated Turkish versions. The final version of the translation and the original scale were submitted to expert reviewers for validity evaluation. The questionnaire was finalized in accordance with the The varimax rotation method and principal component analysis were applied to determine the factor structure of the APAIS. Factorial analysis revealed a two-factor structure with eigenvalues of more than 1.00 that explained 81% of the total variance. Examining the factor structure of the APAIS revealed that the first factor explained 48.98% of the variance, the second factor explained 32.45% of the variance, and all of the factors explained 81.43% of the total variance. The loads of items 1, 2, 4, and 5 on the anxiety subscale ranged between 0.75 and 0.91 and those of items 3 and 6 on the information requirement subscale were 0.90 and 0.92. The two subscales of the APAIS showed high reliability (anxiety α = 0. 89 and information requirement α = 0.78) (Table 3). 

**Table 2:** Internal consistency and homogeneity of personal report of Amsterdam Preoperative Anxiety and Information Scale (APAIS).

Items	Average of scale if item is removed	Variance of scale if the item is removed	Corrected item-total correlation	Cronbach alpha coefficient of the scale if the item is removed
1	11.05	24.21	0.699	0.849
2	11.23	24.55	0.742	0.843
3	10.78	24.61	0.654	0.871
4	11.08	24.43	0.696	0.850
5	11.04	23.85	0.734	0.843
6	10.61	23.73	0.639	0.860

Spearman’s coefficient is used to measure the strength and the direction of monotonic association between two variables. The correlation between the APAIS and the STAI-I was higher than that between the APAIS and STAI-II (Table 4). This relationship showed that the same characteristic could be explored using the APAIS and STAI-I. 

**Table 3:** Factor structure, exploratory variance values, and eigenvalues of the scale.

Factors	Items	Cronbach alpha	Factor loadings
Factor 1	1. I am worried about the anesthesia. 2. The anesthesia is constantly on my mind 4. I am worried about the procedure. 5. The procedure is constantly on my mind.	0.897	0.911
			0.814
			0.880
			0.752
Factor 2	3. I would like to know as much as possible about the anesthesia.	0.786	0.928
	6. I would like to know as much as possible about the procedure.		0.900
Total Cronbach alpha		0.874	
	Exploratory variance values of factors	Eigenvalues	
Factor 1	48.984	3.741	
Factor 2	32.452	1.145	
Total variance 81.435%			

## 4. Discussion

The validity and reliability of the translated APAIS was evaluated in accordance with the principles stated in the related literature (23,25–27). First, linguistic validity was confirmed. To minimize differences, to carefully examine scale items, to transform the meaning of the language in the language in which it is translated, and to standardize the individuals who use this language according to norms provides a basis in adapting the scale into a new culture (23). In this study, the back-translation method was used and the scale was translated in accordance with the literature by expert researchers who knew both languages and the properties of both cultures. In accordance with the opinions of the experts, the language validity of the scale was approved. A good factor analysis requires the KMO value to be equal to or greater than 0.70 (23,24). In this study, the KMO value indicates that we have obtained a sufficient sample for this study.

Item analysis refers to the analysis of the relationship between the value of each item of the measurement tool and the total value of the whole measurement tool. Item value and total value are expected to be highly related if the items of the measurement tool are of equal weight and independent of each other. Scale items with low coefficients are insufficiently reliable. An item–total correlation coefficient of less than 0.25 is indicative of insufficient reliability (28). The item–total correlation scores of the Turkish version of the APAIS are between 0.843 and 0.871 points (Table 2). The item–total score correlation coefficients of all the items exceed 0.250. Therefore, the item–total correlation values ​​of the Turkish version of the APAIS are at the appropriate confidence level.

**Table 4:** Spearman’s coefficients for the APAIS with STAI.

	APAIS	P-value
STAI -I	0.466	0.000
STAI -II	0.369	0.000
STAI total	0.473	0.000

Cronbach’s α-coefficient must exceed 0.70 to ensure the internal consistency of the scale (29). Cronbach’s α-coefficient was calculated to validate the internal consistency and homogeneity of the Turkish version of the APAIS. Cronbach’s alpha coefficients of the entire scale and subscale were used to determine the reliability of the scale. There were similarities between this study and the original scale created by Moerman et al. (11). There were also similarities between the APAIS Cronbach’s alpha coefficients found by studies on the Japanese (13), Italian (14), German (15), and Turkish APAIS. The anxiety subscale has a higher Cronbach’s alpha value than the information requirement subscale. This subscale also has higher Cronbach’s alpha coefficients in the original scale by Moerman et al. (11) and the Japanese scale by Nishimori et al. (13), Italian scale Buonanno et al. (14), and German scale by Berth et al. (15). This finding gives rise to the thought that patients waiting for surgery in Turkey and in other societies have similar perceptions in terms of preoperative anxiety.

Construct validity is another criterion for testing the validity of measurement tools. The literature states that items with a factor load of less than 0.300 should be removed (29–32). The factor loadings of the Turkish version of the APAIS range from 0.752 to 0.928 (Table 3). Therefore, no scale item was removed. The factor structure obtained through factorial analysis indicates that the Turkish version of the APAIS has construct validity. The construct validity of the Turkish version of the APAIS is similar to that of the original scale (11). Test–retest reliability was assessed to determine the time invariance of the scale and revealed that the first and second application of the Turkish version of the APAIS are positively and significantly correlated (r = 0.990; P < 0.000). This finding indicates that the scale can be used reliably (22,26,30). To test external validity, the Turkish version of the APAIS was compared with STAI-I and STAI-II. The APAIS and STAI-I have the same characteristics (Table 4). STAI-I and the original and Italian versions of the scale are correlated (11,14). 

In conclusion, the results of the validity and reliability analyses conducted in this study indicate that the Turkish version of the APAIS can be used to simply and quickly detect the presence and severity of symptoms of preoperative anxiety and the requirement for information. It may be a useful alternative for measuring the preoperative anxiety levels of patients who will undergo elective surgery. The Turkish version of the APAIS with two subdimensions is a suitable alternative to the original scale. It is a valid and reliable instrument for the measurement of preoperative anxiety and information requirements among Turkish patients and possesses the same scale structure as that possessed by the original Dutch version.
